# Preliminary hysteroscopic tubal hydrotubation improves fertility outcomes after laparoscopic salpingotomy for tubal ampullary pregnancy

**DOI:** 10.1186/s12893-021-01367-0

**Published:** 2022-02-12

**Authors:** Qing Wu, Yanling Lin, Jing Zhang, Yun Zhou, Lifeng Chen, Tan Lin

**Affiliations:** 1 Department of Gynecology, Reproductive Medicine Center, Zhejiang Provincial People’s Hospital, Affiliated People’s Hospital, Hangzhou Medical College, Hangzhou, 310014 Zhejiang People’s Republic of China; 2 Department of Obstetrics and Gynecology, Fujian Provincial Hospital, Clinical Medical School of Fujian Medical University, 134, East Road, Fuzhou, 350001 Fujian People’s Republic of China

**Keywords:** Laparoscopy, Ampullary pregnancy, Tubal hydrotubation, Fertility

## Abstract

**Background:**

Salpingotomy may change the anatomical structure and patency of the fallopian tube, which may affect the fallopian function. This study is to investigate the clinical efficacy of preliminary hysteroscopic tubal hydrotubation (HTH) after laparoscopic salpingotomy for tubal ampullary pregnancy.

**Methods:**

A retrospective study was carried out, 140 women underwent laparoscopic salpingotomy for ampullary pregnancy from March 2013 to March 2017. Some patients received HTH in the 1st month and hysterosalpingography (HSG) in the 3rd month after salpingotomy (HTH group; n = 95), and some patients only received HSG in the 3rd month after salpingotomy (control group; n = 45). Clinical data, tubal patency and fertility outcome were evaluated after follow-up of 15 months.

**Results:**

The tubal patency rate of the operated side was significantly higher in the HTH group than that in the control group (89.47% vs 68.89%, *P* < 0.05). The intrauterine pregnancy (IUP) rate was significantly higher in the HTH group (76.47% vs 51.11%, *P* < 0.05), and the recurrent ectopic pregnancy rate in the operated side was significantly lower in the HTH group than in the control group (9.41% vs 22.22%, *P* < 0.05). Logistic regression analysis showed that the positive factor for IUP was HTH (OR = 3.109, 95% CI 1.439–6.714, *P* = 0.004), while the negative factors were history of pelvic inflammatory disease (PID) (OR = 0.167, 95% CI 0.074–0.377, *P* < 0.001) and history of tubal infertility (OR = 0.286, 95% CI 0.113–0.723, *P* < 0.05).

**Conclusion:**

Preliminary HTH after laparoscopic salpingotomy for ampullary pregnancy could improve reproductive function and lead to a better fertility outcome. Patients without history of PID or tubal infertility may be the most suitable ones for HTH after salpingotomy.

## Background

Ectopic pregnancy (EP) occurs approximately 1–2% of all pregnancies [1]. Approximately 98% of ectopic pregnancies occur in the fallopian tube, and the ampullary portion is the most common site of implantation [2]. EP is a fertility-related problem for the reproductive-aged women desiring a future pregnancy [3]. Currently, laparoscopic surgery is the preferred treatment [4]. There are two types of surgical procedures for tubal pregnancy: the radical approach (salpingectomy) and the conservative approach (typically salpingotomy). Salpingectomy has been the standard procedure to remove ectopic pregnancy until laparoscopic salpingotomy was first introduced in 1993 by Bruhat et al. [5]

Many patients who desire to have children in future consider conservative surgery as the optimal treatment. Unfortunately, patients with salpingotomy can be at high risk of recurrent ectopic pregnancy (REP) and secondary infertility later on [6]. What’s more, there is an increased incidence of persistent ectopic pregnancy (PEP) [7] in these patients.

At present, rare progress has been made to overcome the disadvantages of salpingotomy and reduce corresponding complications. In this study, the hysteroscopic tubal hydrotubation (HTH) was hypothesized to be a beneficial method for the recovery of fallopian function and anatomical structure after salpingotomy. In our hospital, all patients were suggested to received HTH in the 1st month and hysterosalpingography (HSG) in the 3rd month after salpingotomy, while some patients rejected HTH and only received HSG in the 3rd month after salpingotomy to evaluate the patency of fallopian. We retrospectively analyzed data on the tubal patency and the clinical reproductive outcomes of these two groups to investigate the clinical efficacy of preliminary HTH.

## Material and methods

### Study subjects

140 tubal ampullary pregnancy patients who received laparoscopic salpingotomy in the Department of Obstetrics and Gynecology in Fujian provincial hospital from March 2013 to March 2017 were enrolled. The study was approved by the board of Fujian provincial hospital ethics committee (No. 201400032), and preoperative informed consent was obtained from all patients after providing explanations of the possible risks and complications.

The following conditions were used as inclusion criteria for laparoscopic salpingotomy and this study: a visible ectopic ampullary tubal mass of natural conception by transvaginal sonography; preoperative serum β-hCG less than 10,000 mIU/ml, maximal diameter of ectopic mass less than 5 cm, a desire to maintain optimal tubal patency for future fertility; a minimum age of 18 years; an appropriate medical status for laparoscopic salpingotomy; and consent to surgical treatment and follow-up. Patients with recurrent tubal pregnancy, gynecologic malignancy, nontubal infertility diseases, incomplete records or follow-up were excluded.

### Surgical procedures

All operations were performed by the same group of surgeons. Patients were placed in the trendelenburg position. In all cases, a 10-mm trocar was inserted in the umbilicus, and three 5-mm trocars were inserted in the lower abdomen. A dilute solution of vasopressin (3 U vasopressin in 10 ml saline solution) was injected into the mesosalpinx around the trouble tube to reduce blood loss. Salpingotomy involved a linear incision performed with scissors on the most prominent part of the fallopian tube, where correlated to the EP location. The ectopic mass was removed by the combination use of hydrodissection and traction with atraumatic forceps. Aspiration and compression lateral to the incision site were applied to facilitate the removal of the products, if necessary. The fallopian tube was closed in a single layer by two or three interrupted stitches using 3-0 VICRYL (Ethicon, USA). In all patients, methotrexate (MTX) (1 mg/kg) was injected locally into the mesosalpinx near the EP site.

### Hysteroscopic tubal hydrotubation (HTH)

Some patients received HTH in the 1st month after salpingotomy. Surgery was performed within 3–7 day of obvious menstruation, and sex was prohibited for the remaining menstrual cycle. The bladder was emptied before surgery, and those patients with acute pelvic inflammatory disease (PID), vaginitis, or other contraindications were excluded. The cervix was grasped with a tenaculum and dilated to accommodate the 7-mm hysteroscope. The scope in the uterine cavity was advanced under direct visualization. The artificial plastic catheter was inserted about 1 mm into the uterotubal junction under hysteroscopy. Hydrotubation was carried out with a solution containing normal saline 20–40 ml + chymotrypsin 1500 U + dexamethasone 5 mg + methylthioninium chloride injection (2 ml:20 mg) 0.1 ml under 20-kPa pushing pressure monitored by ultrasound scanning. The solution dispersed into the pelvic cavity if the tubes were unobtrusive and could be recanalized by hydrotubation.

### Hysterosalpingography (HSG)

All patients received HSG in the 3rd month after laparoscopic salpingotomy. Surgery was performed within 3–7 day of obvious menstruation. HSG was performed in a standard sterile technique. The patient was placed in a lithotomy position, and a vaginal speculum was inserted. After cleaning the external os with povidone-iodine solution, the cervical os was cannulated with a balloon catheter. A cervical tenaculum was not used. The balloon catheter was inflated within the endocervical canal or lower uterine cavity, and contrast injection was performed with nonionic contrast medium iohexol injections, 20 ml/6 g/branch (Beijing Hokuriku Pharmaceutical Co., Ltd., Beijing, China). The radiologists remotely administered the bolus injection using an automatic injection of 30% iohexol contrast agent at a volume of approximately 15 ml, according to the injection pressure and the patient’s response to the regulated injection rate. The entire process was observed under timely and accurate radiography. Images of early and maximal opacification of the uterine cavity, fallopian tubes, and peritoneal contrast spillage were obtained. Fifteen minutes of photographic pelvic diffusion was recorded.

Fallopian tubal patency was assessed by HSG. The tubal patency was classified into three diagnostic grades: (a) patent, if the dye was seen in the whole tube during the injection, then totally disappeared from the tube but appeared in the pelvis 15 min later; (b) passable, if the dye was seen from the cornu to fimbria ends during the injection and more than two-thirds of the dye disappeared from the tube but was seen in the pelvis 15 min later; and (c) completely blocked, if the dye was not seen from the tubal cornu or no dye was seen in the pelvis 15 min later [8].

Patients were followed for up to 15 months after laparoscopic surgery. Data were analyzed to obtain a reproductive estimation. Intrauterine pregnancy (IUP) was verified by an ultrasound scans showing a fetal pole with a heartbeat. REP of the operated tube was diagnosed by laparoscopy and pathology, and secondary infertility was determined by performing interviews to assess if the patient failed to conceive for 1 year after HSG.

### Statistical analysis

All statistical analyses were performed with SPSS 21.0 software (IBM, USA). Continuous variables were recorded as means ± SDs. Categorical variables were described using proportions. Baseline patient characteristics were calculated via t-test for comparisons of normally distributed data and the rank-sum test for comparisons of non-normally distributed data. Count data were summarized as percentages and compared using the Chi-square test and Fisher’s exact tests. Multivariate logistic regression was used to evaluate the relative factors. A P value < 0.05 was considered statistically significant.

## Results

All patients received HSG in the 3rd month after salpingotomy for future pregnancy. Based on whether patients received HTH in the 1st month after the operation, the patients were assigned into two groups: the HTH group (n = 95) underwent HTH in the 1st month and HSG in the 3rd month after the operation, and the control group (n = 45) received only HSG in the 3rd month after salpingotomy.

The demographic clinical characteristics of the patients are displayed in Table 1. The mean ages were 28.34 ± 3.56 years and 27.98 ± 3.72 years in both groups, respectively (*P* = 0.875). There were no significant differences in gravidity, parity, history of PID or history of tubal infertility between the two groups (*P* > 0.05). The mean preoperative serum β-hCG levels of the two groups were 3488 ± 633.82 mIU/ml and 3415 ± 617.88 mIU/ml, and the mean durations of gestation were 43.82 ± 2.77 days and 43.44 ± 2.78 days (*P* > 0.05). The mean operative time and volume of bleeding were not significantly different. All ectopic pregnancies were confirmed by pathology. The β-hCG levels of all the patients in each group returned to baseline in less than 4 weeks after surgery. There were no procedural complications, such as reproductive systemic infection.

**Table 1:** Baseline characteristics (n = 140)

Characteristics	HTH group (n = 95)	Control group (n = 45)	*P*
Age (years)	28.3 ± 3.6	28.0 ± 3.7	0.875
Gravidity (n)	34	18	0.630
Parity (n)	16	6	0.594
History of PID	22	16	0.123
History of tubal infertility (n)	14	9	0.433
Preoperative serum β-hCG (mIU/ml)	3488.0 ± 633.8	3415.0 ± 617.9	0.564
Duration of gestation (days)	43.8 ± 2.8	43.4 ± 2.8	0.992
Maximal diameter of ectopic mass (cm)	3.1 ± 0.5	3.0 ± 0.4	0.237
Operative time (min)	49.7 ± 7.7	49.4 ± 7.7	0.157
Volume of bleeding (ml)	16.8 ± 4.9	17.9 ± 5.8	0.933

The patency rates of the operated tube after operation were 89.47% in the HTH group and 68.89% in the control group respectively, and the difference between the two groups was statistically significant (χ^2^ = 9.109; *P* = 0.003) (Table 2). The patency rates of the contralateral side tube were 88.42% in the HTH group and 82.22% in the control group, and the difference between the two groups was not significant (*P* = 0.317) ).

**Table 2:** The operated tubal patency rate of the two groups 3 months after the operation

	n	Patent (%)	Passable (%)	Completely blocked (%)
HTH group	95	85 (89.47)	7 (7.37)	3 (3.16)
Control group	45	31 (68.89)	8 (17.78)	6 (13.33)
*P*		0.003	0.063	0.022

Ten patients in the HTH group dropped out to conceive after HSG. In the 15th month after surgery, the IUP rates were 76.47% in the HTH group and 51.11% in the control group (χ^2^ = 8.652; *P* = 0.003). The REP rates of the operated side were 9.41% in the HTH group and 22.22% in the control group (χ^2^ = 4.048; *P* = 0.04). There were significant differences (*P* < 0.05) in the IUP rate and REP rate between the two groups. The secondary infertility rates were 14.11% and 26.67% in the HTH and control groups, respectively, without a significant difference (χ^2^ = 3.078; *P* = 0.08) (Table 3).

**Table 3:** IUP rate, REP rate and secondary infertility rate during the 15 months after the surgery

	n	IUP rate (%)	REP rate (%)	Secondary infertility rate (%)
HTH group	85	65 (76.47)	8 (9.41)	12 (14.11)
Control group	45	23 (51.11)	10 (22.22)	12 (26.67)
*P*		0.003	0.044	0.079

Multivariable analysis analyzed the influence factors for fertility outcome was listed in Table 4. Logistic regression analysis showed that the positive factor for IUP was HTH (odds ratio [OR] = 3.109, 95% confidence interval [CI] 1.439–6.714 *P* = 0.004), while the negative factors were history of PID (OR = 0.167, 95% CI 0.074–0.377 *P* < 0.001) and history of tubal infertility (OR = 0.286 95% CI 0.113–0.723 *P* < 0.05).

**Table 4:** Logistic regression analysis discussing relative factors for fertility outcome

	OR	95% CI	*P*
HTH	3.109	1.439–6.714	0.004
History of abortion	0.725	0.306–1.714	0.464
History of PID	0.167	0.074–0.377	0.000
History of tubal infertility	0.286	0.113–0.723	0.008

## Discussion

EP is a severe gynecological problem among reproductive-aged women. In recent years, laparoscopy has become preferable to laparotomy for the treatment of tubal EP [9]. In clinical practice, there are two types of surgical procedures for tubal pregnancy: salpingectomy and salpingotomy. The choice of salpingotomy versus salpingectomy depends on many factors, including the age of the patient, the tube condition, the serum human chorionic gonadotropin levels, the diameter of the tubal mass and the patient’s future fertility desire.

The advantages and disadvantages of salpingotomy for EP have been debated for many years [10, 11]. Although ectopic pregnancy lesions are conservatively cleared by salpingotomy, the change of the anatomical structure and patency of the fallopian tube may affect the fallopian function, resulting in decreased fecundity [12].

Salpingotomy also has several disadvantages. One disadvantage of salpingotomy is that it may increase the risk of PEP. A large tubal mass in EP is a high-risk factor for PEP after laparoscopic salpingotomy; therefore, β-hCG monitoring is mandatory [13]. Several studies reported that the rate of persistent trophoblastic disease was reduced by the use of prophylactic MTX in laparoscopic salpingotomy, which could be administered by local MTX injection into the mesosalpinx near the site of the EP [12, 14]. In this study, all patients were injected with local MTX, and no patients suffered from PEP after laparoscopic salpingotomy.

Women with previous EP have higher incidence of REP and miscarriage in their second pregnancies [13]. Infertility and EP commonly occur due to causes such as tubal obstruction and pelvic adhesion [15]. Some studies compared subsequent fertility after salpingectomy to that after salpingotomy and found no statistically significant differences in the fertility outcomes [16, 17]. Turan et al. reported no significant differences in IUP rates up to 24 months after surgical treatment in younger patients undergoing salpingectomy compared to those undergoing salpingotomy [18]. However, several studies reported conflicting findings that there were higher risks for REP or lower cumulative IUP rates after laparoscopic salpingotomy [9, 19, 20]. Bennetot et al. reported that the 24-month cumulative rate of IUP was 76% after medical treatment, 67% after conservative surgery, and 67% after radical salpingostomy. The corresponding REP rates were 25.5%, 18.5%, and 18.5% within 2 years, respectively [21]. In this study, the REP rate in the HTH group was 9.41%, which was even lower than that reported result, and the 12-month cumulative rate of IUP is 76.47% in the HTH groups. In this study, logistic regression analysis showed that the positive factor for IUP was HTH, while the negative factors were history of PID and history of tubal infertility.

As the structure and function of the fallopian tube need to be rebuilt after laparoscopic salpingotomy, it would be great to have an effective approach which can improve the recovery of fallopian and pelvic function. Recently, hysteroscopic techniques are highly accurate and sensitive for detecting fallopian tubal obstruction [22]. Therefore, the HTH was hypothesized as a beneficial approach for the recovery of fallopian. To our knowledge, this is the first study on the efficacy of HTH after salpingotomy with the aim of improving the patency of the fallopian tube and reducing complications.

In this study, HTH was performed 3–7 day after the first postsurgical menses in 95 patients. This procedure provided an opportunity not only to check the tubal patency and uterine cavity but also to assist fluid going through the inner cavity of the fallopian tube with hydrotubation. We hypothesized that tubal hydrotubation after salpingotomy could reduce blood coagulation and inflammatory factors, which may prompt the recovery of fallopian function and anatomical structure. Besides, the drugs used in hydrotubation are anti-inflammatory and may promote the recovery of oviduct. Lei et al. reported that hysteroscopic hydrotubation solution consisted of hydrocortisone (20 mg), gentamicin (160,000 IU) and procaine (80 mg) in 20 ml distilled water and treated the tubal blockage [23]. Our study also shows that patients who have undergone HTH have better potential fallopian and fertility outcomes. However, this study still has some shortcomings. Firstly, it is a retrospective cohort study with a limited sample size. Secondly, it is necessary to organize a prospective randomized controlled trial with multiple centers to confirm the results. As all surgical procedures were performed by the same surgery group in this study, the generalizability of the results may be weakened. Moreover, the mechanism of fallopian recovery should be further studied by animal models and clinical trial, especially, the mechanism of promoting effect of hysteroscopic tubal hydrotubation should be investigated.

## Conclusion

This study indicated that the tube-preserving surgery by salpingotomy followed by HTH could represent an option for ectopic tubal pregnancy in women with a strong desire for fertility. Besides, based on the results of logistic regression analysis in this study, patients without history of PID or tubal infertility might be the most suitable ones for HTH after salpingotomy, which could achieve the most satisfactory fertility outcome.

## Data Availability

The data of study are not publicly available due to ethical and legal restrictions. However, upon request, data may be available from the corresponding author on reasonable request.

## Abbreviations

HTH: Hysteroscopic tubal hydrotubation
EP: Ectopic pregnancy
REP: Recurrent ectopic pregnancy
PEP: Persistent ectopic pregnancy
HSG: Hysterosalpingography
MTX: Methotrexate
PID: Pelvic inflammatory disease
IUP: Intrauterine pregnancy
